# Supplementary material for: Development of a prediction model for lower limb deep vein thrombosis in critically ill patients after intracranial hemorrhage at high altitude: a retrospective study
Source: PeerJ. 2025 Nov 13;13:e20245. doi: 10.7717/peerj.20245 (PMC12619939; doi:10.7717/peerj.20245)
Supplement: Supplemental Information 1 [file peerj-13-20245-s001.docx]

**1. Hypertension-related Intracerebral Hemorrhage (HICH)**

Goals: Hematoma evacuation, intracranial pressure (ICP) reduction, neurological function preservation.

**Basal Ganglia Hemorrhage:**

Hematoma volume ≥30 mL and progressive neurological deterioration or coma.

Ventricular extension with hydrocephalus requires concurrent ventriculostomy.

**Thalamic Hemorrhage:**

Hematoma volume ≥15 mL with hydrocephalus or progressive neurological deficit.

**Cerebellar Hemorrhage:**

Hematoma volume ≥10 mL or diameter ≥3 cm with brainstem compression or hydrocephalus.

Urgent surgery indicated even with smaller volumes if consciousness deteriorates or hydrocephalus develops.

**Lobar Hemorrhage:**

Hematoma volume ≥30 mL, especially in superficial locations accessible for surgical resection.

**Intraventricular Hemorrhage (IVH):**

Severe hydrocephalus with altered consciousness → urgent external ventricular drainage (EVD).

Consciousness Status:

Patients with Glasgow Coma Scale (GCS) 9–12 (moderate coma) and surgically accessible hematoma may benefit.

For GCS ≤8 (severe coma), surgery is considered cautiously, preferably in young patients with early signs of herniation.

Dynamic Changes:

Progressive neurological deterioration (e.g., pupillary changes, hypertension, respiratory abnormalities).

Imaging evidence of hematoma expansion (≥30% volume increase or ≥6 mL absolute increase).

**2. Subarachnoid Hemorrhage (SAH, Aneurysmal):**

Goals: Aneurysm obliteration, subarachnoid blood clearance, prevention of vasospasm.

**Aneurysm:**

All ruptured aneurysms require urgent intervention (clipping or endovascular coiling), particularly:

Hunt-Hess Grade I–III (mild to moderate neurological deficits).

Selected Grade IV patients with stable systemic conditions.

Unruptured aneurysms: Consider intervention for those ≥5 mm in diameter, irregular morphology, or high-risk locations.

**Hydrocephalus:**

Acute hydrocephalus with altered consciousness → EVD.

Delayed Cerebral Ischemia:

Refractory cerebral vasospasm despite medical treatment → endovascular therapy (e.g., balloon angioplasty).

**3. Subdural Hematoma (SDH):**

**Acute SDH:**

Surgical evacuation indicated for:

Hematoma thickness ≥10 mm or midline shift ≥5 mm, regardless of consciousness level.

Hematoma thickness <10 mm and midline shift <5 mm but GCS <9 or hematoma progression on imaging.

**Chronic SDH:**

Surgical drainage (burr hole or craniotomy) for:

Neurological symptoms (e.g., hemiparesis, aphasia, altered consciousness).

Progressive hematoma enlargement or mass effect (thickness ≥15 mm or midline shift ≥5 mm).

**4. Epidural Hematoma (EDH):**

**Urgent craniotomy for:**

Progressive consciousness deterioration or focal neurological signs (e.g., hemiparesis, pupillary dilation).

Hematoma volume ≥30 mL, thickness ≥15 mm, or midline shift ≥5 mm.

Temporal EDH ≥20 mL (high risk of transtentorial herniation due to proximity to brainstem).

**5. Special Considerations:**

Geriatric Patients: Balance surgical risk with comorbidities (e.g., cardiopulmonary dysfunction).

Coagulopathy: Correct coagulation abnormalities (e.g., vitamin K, fresh frozen plasma) before surgery to minimize rebleeding risk.

Pediatric Patients: Lower volume thresholds for intervention, prioritizing neurological preservation.

Contraindications:

Advanced brain herniation (bilateral fixed dilated pupils, decerebrate posturing) with GCS ≤3 (poor prognosis).

Severe systemic comorbidities (e.g., end-stage organ failure) precluding anesthesia.

Stable clinical condition with minimal mass effect and expected hematoma resolution.
